# How should we deal with misattributed paternity? A survey of lay public attitudes

**DOI:** 10.1080/23294515.2017.1378751

**Published:** 2017-10-18

**Authors:** Georgia Lowe, Jonathan Pugh, Guy Kahane, Louise Corben, Sharon Lewis, Martin Delatycki, Julian Savulescu

**Affiliations:** a Bruce Lefroy Centre for Genetic Health Research Murdoch Children's Research Institute and Department of Paediatrics, University of Melbourne; b Uehiro Centre for Practical Ethics, University of Oxford; c Bruce Lefroy Centre for Genetic Health Research, Murdoch Children's Research Institute and School of Psychological Sciences, Monash University; d Bruce Lefroy Centre for Genetic Health Research, Murdoch Children's Research Institute and Public Health Genetics, Murdoch Children's Research Institute; e Bruce Lefroy Centre for Genetic Health Research, Murdoch Children's Research Institute, Department of Paediatrics, University of Melbourne, School of Psychological Sciences, Monash University, and Clinical Genetics, Austin Health

**Keywords:** ethics, genetics, misattributed paternity, practice guidelines as a topic, public perspective, questionnaire

## Abstract

**Background**: Increasing use of genetic technologies in clinical and research settings increases the potential for misattributed paternity to be identified. Yet existing guidance from the President's Commission for the Study of Ethical Problems in Biomedical and Behavioral Research and the Institute of Medicine (among others) offers contradictory advice. Genetic health professionals are thus likely to vary in their practice when misattributed paternity is identified, and empirical investigation into the disclosure of misattributed paternity is scarce. Given the relevance of this ethical dilemma and its significance to users of genetic services, this study aimed to investigate the attitudes of lay people with regard to the disclosure of misattributed paternity. **Methods**: An online questionnaire was hosted and advertised through Amazon's Mechanical Turk to 200 United States residents aged 18 years or older. Respondents were asked to rate (via a Likert scale) the ethical permissibility of possible actions a clinician may carry out when misattributed paternity is identified. Data analysis consisted of preliminary descriptive analysis, chi-squared analysis, and Wilcoxon signed-rank tests. **Results**: There was no clear majority support for many of the options surveyed across different contexts, with only six out of ten scenarios displaying some general consensus. Men were more likely to support scenarios where the father is informed of paternity. Importantly, participants' views varied according to whether the desires of the father were previously expressed, suggesting that perceptions of the permissibility of a clinician's action will depend on the interests of all parties affected. **Conclusions**: This sample of the general public showed attitudes that were, at least to some degree, at variance with some professional guidelines. We give arguments for why at least some of these attitudes might be justified. We argue that case-specific judgments should be made and outline some of the relevant ethical considerations. While general guidelines ought to be considered, context-specific moral judgments cannot be avoided.

## Background

With continuing advances in genetic technologies, clinicians have greater access than ever before to using these genetic technologies in diagnostic medicine. The revolution of advanced genetic and genomic technologies in clinical settings has increased the potential for incidental findings coming to light. Incidental findings are those findings that are unanticipated or unrelated to the original purpose for which the testing occurred (Wolf et al. 2008).

Misattributed paternity, where the assumed father is not the biological father, is an incidental finding that is encountered by clinicians conducting genetic testing in families or in the genetic research setting. A recent study established that of 102 genetic health professionals, 35 had encountered misattributed paternity (McLean et al. 2013). Rates of misattributed paternity vary between studies, ranging from 0.8% to 30% (median 3.7%, *n* = 17) (Bellis et al. 2005). The large discrepancy in the rate of misattributed paternity is thought to be due to nonrepresentative populations in some studies, and thus attributed to cultural and societal factors (Bellis et al. 2005).

Existing guidelines about how to handle the discovery of misattributed paternity offer contradictory advice (Hercher and Jamal 2016), and this may plausibly lead a genetic health professional to vary considerably in his or her practice (Hope, Savulescu, and Hendrick 2008). For instance, Hercher and Jamal (2016) point out that the 1983 President's Commission for the Study of Ethical Problems in Biomedical and Behavioral Research recommended that misattributed paternity be disclosed to both the woman and her partner, while the Institute of Medicine (IOM) committee on assessing genetic risks advocated informing the mother, but not her partner, about misattributed paternity (Institute of Medicine 1994).

Although it has been argued that incidental findings should not be reported at all unless they have clinical significance (Berg, Khoury, and Evans 2011), genetic specialists’ perspectives are divided on this point (Downing et al. 2013). However, existing studies suggest that most genetic health professionals are generally against disclosure to the assumed father (Pencarinha et al. 1992; Turney 2005; Wertz, Fletcher, and Mulvihill 1990). In support of this view, it has been argued that (i) disclosure of misattributed paternity breaches the duty of confidentiality to the mother and the mother's right to privacy; (ii) it breaches the duty of nonmaleficence to at least one parent and the child through potentially disrupting family harmony; and (iii) misattributed paternity is an incidental finding and there is therefore no duty to inform (Lucassen and Parker 2001; Lucast 2007; Schroder 2009; Turney 2005; Wright et al. 2002).

Yet this view has been challenged by some medical ethicists, who have highlighted (i) the importance of this information to the child and presumed father's autonomy, (ii) a general duty for doctors to disclose material information, (iii) the implications that nondisclosure may have for the erosion of trust in medical professionals, and (iv) the claim that it is paternalistic of clinicians to not disclose through making value judgments on what they feel is in the best interests of the family (Lucassen and Parker 2001; Lucast 2007; Ross 1996; Schroder 2009; Turney 2005; Wright et al. 2002).

Given this ongoing ethical debate, it is surprising that there is a lack of empirical data regarding the attitudes of both service users and lay public attitudes toward the disclosure of misattributed paternity. We believe that such data are important to the ethical debate surrounding disclosure of misattributed paternity. First, although the attitudes of service users and the lay public should not be the sole determinant of the right course of action in these situations, ethical norms should be sensitive to the views of both service users (individuals who currently use medical facilities) and members of the lay public who not only may fund these facilities through taxes, but may require these facilities in the future. Second, by investigating lay public attitudes, we may identify a wider range of concerns and factors that may enter into ethical decision making in this area than are currently discussed in the academic literature. While such concerns do not directly dictate answers to the ethical question, they can help widen the scope of the reflective equilibrium methodology employed by coherentist approaches to empirical bioethics (Davies, Ives, and Dunn 2015). In this way, empirical data about lay attitudes can usefully inform and broaden bioethical analysis (Strong, Lipworth, and Kerridge 2010).

Only one prior study has addressed the views of the lay public regarding dislosure of misattributed paternity (Turney 2005). In this national study (which was given detailed follow-up in selective focus groups), 1000 random Australian participants were asked how comfortable they were, on a scale of 1–10, “providing DNA results to a man—where the man and his family have had a DNA test for an inherited disease—but the test shows he is not actually the father of one of the children.” Turney (2005) found that respondents reported a slightly higher than average level of comfort (*M* = 6.5) with disclosure to the father. Although level of education had a significant main effect on comfort with revealing false paternity to the father (with lower educated respondents expressing greater comfort with telling the presumed father about his nonpaternity than those with a relatively higher education), no significant effects were found for any other demographic, including gender.

Due to increased clinical use of genetic technologies, further empricial research into the management of misattributed paternity is required. This article will supplement Turney's (2005) pioneering study of lay public attitudes to misattributed paternity in four ways. First, it will provide data about lay attitudes toward misattributed paternity among a U.S. population, which may contrast with Turney's (2005) Australian sample. Second, our survey will provide new data about lay attitudes toward a range of potential courses of action a doctor could take in a situation of an incidental finding indicating misattributed paternity. Third, our survey will provide data about the bearing that the father's expression of a wish to know about potential incidental findings related to paternity may have on lay attitudes toward the different courses of action a doctor might take. Finally, it will provide data about lay attitudes toward carrying out mandatory paternity testing at birth.

## Methods

### Questionnaire

A questionnaire was developed that contained two vignettes regarding misattributed paternity. The questionnaire was reviewed by several experts who have had considerable experience relating to the dilemmas presented in the vignettes, including a clinical geneticist and a general practitioner. Feedback was sought regarding content and validity.

A 7-point Likert scale was used to ascertain respondent views on how ethically permissible the behaviour of the doctor in the given scenario is, with a score of 1 representing completely unacceptable and a score of 7 representing absolutely acceptable. In what follows, in the interests of clarity, we use headings to describe each option. It is important to note that these headings did not appear in the actual survey; rather, each option was assigned a letter for identification.

The two vignettes were as follows:

Vignette 1 described a scenario in which the father is silent on the results of testing:

A married couple bring their son to doctors with an abnormality. During their investigations, the doctors find that the child could not be the son of the man (the husband of the child's mother). **He believes he is the genetic father**. In your view, how ethically permissible would it be for the doctor to choose to do each of the following?

A. DISCUSS—Discuss this confidentially with the mother?
B. IGNORE—Ignore it on the basis that it is not relevant to the child's abnormality?
C. ENCOURAGE—Encourage the mother to reveal this finding to her husband and explain it?
D. INFORM—Inform the husband that he is not the father of the child, even if the woman does not want them to?

Vignette 2 described a scenario where the father makes enquiries relevant to the incidental finding:

A married couple brings their son to doctors with an abnormality. During their investigations, the doctors find that the child could not be the son of the man (the husband of the child's mother). **He believes he is the genetic father. Suppose that the man asked doctors whether they could tell whether the boy is his son**. In your view, how ethically permissible would it be for the doctor to choose to do each of the following?

A) ASK—Ask permission to reveal the truth from the mother?
B) TELL TRUTH—Tell the truth, regardless of the mother's wishes?
C) LIE, IS SON—Lie, and say that the boy is the man's son?
D) LIE, CAN'T TELL—Lie, and say they can't tell whether or not the boy is the man's son?
E) INFORM—Tell the truth only if the mother lets them?
F) DOCTOR DECIDES—Do what the doctors judge to be in the best interests of the boy?

The following question was also asked: “Do you think that all children should be given paternity testing soon after birth and the father informed of the result?” A 6-point Likert scale was used to establish repondent agreement (with a score of 1 representing strongly disagree and a score of 6 representing strongly agree).^1^1While the two vignettes allowed participants to consider a wide and nuanced range of possible responses, this more general question was designed to probe participants’ overall view on the matter in a blunter way. In order to prevent participants’ responses from clustering around a noncommittal midpoint, here we employed a 6-point Likert scale to force participants to endorse either a positive or negative response to this controversial issue.

Table 1 summarizes the vignettes and outlines the code assigned to each scenario. Scenarios are referred to by their assigned code (and additional title) throughout the rest of the article.

**Table 1.** Vignettes.

**Vignette 1**: A married couple bring their son to doctors with an abnormality. During their investigations, the doctors find that the child could not be the son of the man (the husband of the child's mother). He believes he is the genetic father. In your view, how ethically permissible would it be for the doctor to choose to do each of the following?	A. Discuss this confidentially with the mother?	**1A**
	B. Ignore it on the basis that it is not relevant to the child's abnormality?	**1B**
	C. Encourage the mother to reveal this finding to her husband and explain it?	**1C**
	D. Inform the husband that he is not the father of the child, even if the woman does not want them to?	**1D**
**Vignette 2**: A married couple brings their son to doctors with an abnormality. During their investigations, the doctors find that the child could not be the son of the man (the husband of the child's mother). He believes he is the genetic father. Suppose that the man asked doctors whether they could tell whether the boy is his son. In your view, how ethically permissible would it be for the doctor to choose to do each of the following?	A. Ask permission to reveal the truth from the mother?	**2A**
	B. Tell the truth, regardless of the mother's wishes?	**2B**
	C. Lie, and say that the boy is the man's son?	**2C**
	D. Lie, and say they can't tell whether or not the boy is the man's son?	**2D**
	E. Tell the truth only if the mother lets them?	**2E**
	F. Do what the doctors judge to be in the best interests of the boy?	**2F**
**Additional Question**: Do you think that all children should be given paternity testing soon after birth and the father informed of the result?		***3***
***Answer options***:		
***Vignettes 1 and 2***: *Seven point Likert scale – 1 represents completely unacceptable; 7 represents absolutely acceptable*.		
***Additional Question***: *Six point Likert scale – 1 respresents strongly disagree; 6 represents strongly agree*.		

Participant demographic characteristics of gender, age, and highest level of education were also ascertained.

### Sample and recruitment

Amazon's Mechanical Turk, an online platform, was used to advertise and invite U.S. residents to complete the questionnaire. Amazon's Mechanical Turk (accessed from www.mturk.com) is an online crowd-sourcing service that advertises tasks (such as questionnaires) that users can complete in return for compensation. Multiple research studies have used Mechanical Turk to obtain data (Eriksson and Simpson 2010; Suri and Watts 2011). Importantly, studies have illustrated that data obtained via the use of Mechanical Turk are as reliable as data that have been obtained via more traditional methods (Buhrmester, Kwang, and Gosling 2011; Gosling et al. 2004). The questionnaire was restricted to U.S. residents 18 years or older. To ensure quality of the responses obtained, access to the questionnaire was only advertised to those who had obtained a Mechanical Turk “masters” qualification, meaning they had previously demonstrated reliability and accuracy in completing other questionnaires on this online platform. An instructional manipulation check was also included to ensure participant diligence in completing the survey (Oppenheimer, Meyvis, and Davidenko 2009).

### Statistical analysis

For analysis purposes, responses were collapsed into the following categories for the two vignettes: points 1, 2, and 3 were combined to indicate a belief that the act in question was unacceptable, the middle point 4 indicated the respondent was ambivalent toward the acceptability of the act in question, and points 5, 6, and 7 were combined to indicate a belief that the act in question was acceptable. For the paternity testing at birth question, points 1, 2, and 3 and points 4, 5, and 6 were combined to indicate that participants disagreed and agreed, respectively.

Data analysis was conducted using SPSS (Windows, Version 22.0; SPSS, Inc., Chicago, IL). Preliminary descriptive analysis generated frequency data to elicit the description of respondents. Chi-squared analysis was used to assess the degree of associations between responses and demographic variables. In situations where an expected number in the cell of a chi-squared table was less than five, a Fisher's exact test was used. Wilcoxon signed-rank tests were used to assess if there was a difference between responses given by the same participants, for the same or similar scenario, between each of the two vignettes. A *p* value of <.05 was considered statistically significant for all analyses.

### Ethical approval

This study was conducted with the approval of the Social Sciences and Humanities Inter-Divisional Research Ethics Committee at the University of Oxford (ref. no. SSD/CUREC1 A/13-192).

## Results

### Demographics of respondents

Two hundred respondents completed the questionnaire. All respondents answered the instructional manipulation check correctly. All questions were completed by all 200 respondents. Overall, 64.5% of respondents were female, with 61% of all respondents being aged between 25 and 44 years of age. Only 11.5% of participants had an education level of less than college, with all other participants having completed some college or higher. Table 2 provides a detailed breakdown of participant demographic characterisitics.

**Table 2.** Demographic characteristics of the cohort.

Demographic	Category	Number of participants (percentage), *N* = 200
Age (years)	18–24	26 (13.0%)
	25–34	73 (36.5%)
	35–44	49 (24.5%)
	45–54	34 (17.0%)
	55 or older	18 (9.0%)
Gender	Female	129 (64.5%)
	Male	71 (35.5%)
Highest level of education	Less than high school/high school/general educational development	23 (11.5%)
	Some college	63 (31.5%)
	Two-year college degree	29 (14.5%)
	Four-year college degree (BA, BS)	63 (31.5%)
	Master's degree/doctorate degree/professional degree	22 (11.0%)

### Ethical permissibility of the scenarios

Overall in vignette 1, the response deemed most acceptable (73.5% respondents) was 1 A—DISCUSS, in which nonpaternity is discussed confidentially with the mother. The response deemed least acceptable (75.5% respondents) was 1D—INFORM, in which doctors inform the husband even if the mother did not want them to do so. Interestingly, respondents were divided over the acceptability of 1B—IGNORE (whether to ignore the incidental finding) and 1C—ENCOURAGE (whether to encourage the mother to reveal it).

**Figure 1.**
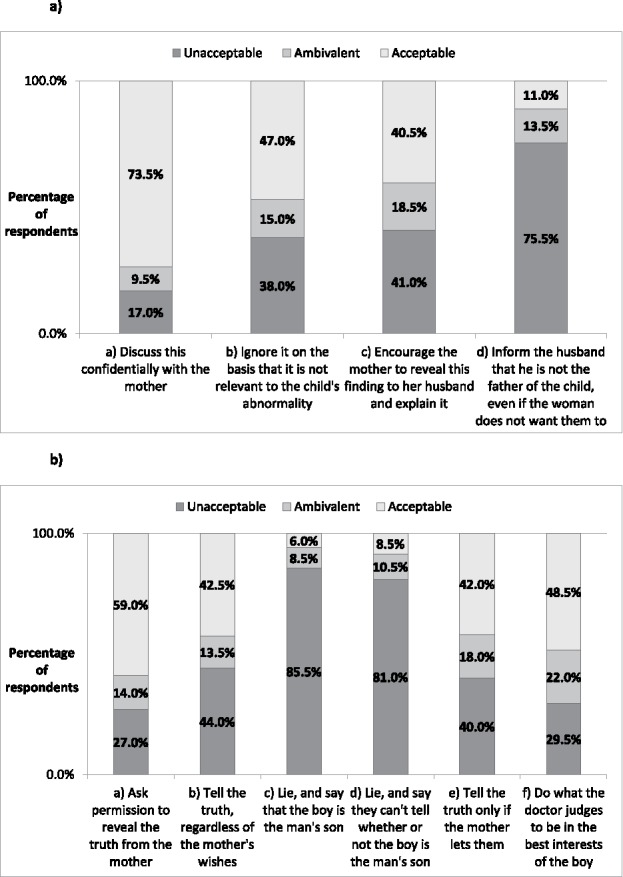
Vignette results showing percentage of respondents for each ethical permissbility category and corresponding doctor scenario. (a) Vignette 1 results. (b) Vignette 2 results.

Overall in vignette 2, in which the father asked doctors whether they could tell whether the boy is his biological son, the response deemed most acceptable (59.0% respondents) was 2A—ASK, in which doctors ask the mother's permission to reveal the truth. The response deemed least acceptable (85.5% respondents) was 2C—LIE, IS SON, in which doctors lie to the man and say the boy is the man's son. Only in the two scenarios involving lying (2C—LIE, IS SON and 2D—LIE, CAN'T TELL) was there strong convergence on rightness and wrongness of that general course of action. Figure 1 outlines results for both vignettes, showing the percentage of respondents for each ethical permissibility category and corresponding scenario. With regard to routine paternity testing at birth (additional question), 51% of respondents disagreed and 49% agreed with this hypothetical concept.

For vignette scenarios 1A—DISCUSS, 2A—ASK, and 2F—DOCTOR DECIDES, responses were skewed to the absolutely acceptable extreme on the Likert scale, while 1D—INFORM, 2C—LIE, IS SON, and 2D—LIE, CAN'T TELL, were skewed to the completely unacceptable extreme on the Likert scale. For vignette scenarios 1B—IGNORE, 1C—ENCOURAGE, 2B—TELL TRUTH, 2E—INFORM, and the routine paternity testing at birth question, responses were not skewed, with a relatively even distribution between the two extremes observed.

A Wilcoxon signed-rank test was used to compare responses given for similar courses of action across the two vignettes. There was evidence of a significant difference in responses from all respondents between the two vignettes with regard to the ethical acceptability of the doctor's informing the father of paternity against the mother's wishes (1D—INFORM and 2B—TELL TRUTH, *Z* = –7.91, *p* < .0005). In vignette 1D—INFORM, 11% of respondents were of the view that disclosing to the man that he is not the biological father even if the mother does not want this to happen was acceptable, while 75.5% of respondents felt this action was unacceptable. However, in scenario 2B—TELL TRUTH (telling the truth regardless of the mother's wishes) a greater proportion of respondents (42.5%) were of the view that this action was acceptable, while a lower proportion of respondents (44%) felt this action was unacceptable.

### Relationship between ethical permissibility and demographic characteristics

Each vignette and corresponding scenario and the paternity testing at birth question were analyzed by chi-squared analyses to ascertain whether there was a relationship between the ethical permissibility of the scenario and each demographic characteristic of gender, age, and education level. Table 3 presents these results.

**Table 3.** Comparison of demographic characteristics and ethical permissibly of scenario.

Vignette and scenario	Demographic characteristic	Significance (chi-squared statistic and *p* value)
1A	Gender	2.17, *p =* .33
	Age	13.47, *p =* .08^b^
	Education	5.69, *p =* .69^b^
1B	Gender	1.50, *p =* .47
	Age	13.17, *p =* .11
	Education	5.69, *p =* .68
1C	Gender	3.72, *p =* .16
	Age	3.87, *p =* .87
	Education	14.86, *p =* .06
1D	Gender	**8.61, *p =* .01**^a^
	Age	5.98, *p =* .65^b^
	Education	4.00, *p =* .87^b^
2A	Gender	1.75, *p =* .42
	Age	8.58, *p =* .37^b^
	Education	11.08, *p =* .20
2B	Gender	**5.80, *p =* .05**^a^
	Age	9.11, *p =* .33
	Education	7.65, *p =* .47
2C	Gender	2.70, *p =* .26
	Age	7.47, *p =* .44^b^
	Education	6.14, *p =* .61^b^
2D	Gender	0.05, *p =* .98
	Age	13.45, *p =* .07^b^
	Education	9.44, *p =* .27^b^
2E	Gender	5.59, *p =* .06
	Age	11.03, *p =* .20
	Education	2.62, *p =* .96
2F	Gender	5.44, *p =* .07
	Age	**18.94, *p =* .02**^a^
	Education	14.00, *p =* .08
3	Gender	**7.41, *p =* .01**^a^
	Age	**9.96, *p =* .04**^a^
	Education	8.31, *p =* .08

a Indicates a *p* value of less than .05 was obtained.

b indicates a Fisher's exact test was used.

Table 4 provides a breakdown of the percentage of respondents for each ethical permissibility category according to demographic characteristic for those scenarios where a *p* value of less than .05 was obtained for analyses between ethical permissibility responses and demographic characteristics.

**Table 4.** Comparison of demographic characteristics and ethical permissibly of scenario with percentage of respondent values.

			Number of participants (% of demographic characteristic)			
			Likert scale			
Question	Demographic	Characteristic	Unacceptable	Ambivalent	Acceptable	Significance
1D	Gender	Male (*n =* 71)	46 (64.8)	16 (22.5)	9 (12.7)	χ^2^ = 8.61, *p =* .01
		Female (*n =* 129)	105 (81.4)	11 (8.5)	13 (10.1)	
2B	Gender	Male (*n =* 71)	24 (33.8)	9 (12.7)	38 (53.5)	χ^2^ = 5.80, *p =* .05
		Female (*n =* 129)	64 (49.6)	18 (14.0)	47 (36.4)	
2F	Age (years)	18–24 (*n =* 26)	3 (11.5)	12 (46.2)	11 (42.3)	χ^2^ = 18.94, *p =* .02
		25–34 (*n =* 73)	26 (35.6)	16 (21.9)	31 (42.5)	
		35–44 (*n =* 49)	18 (36.7)	9 (18.4)	22 (44.9)	
		45–54 (*n =* 34)	9 (26.5)	3 (8.8)	22 (64.7)	
		55 and older (*n =* 18)	3 (16.7)	4 (22.2)	11 (61.1)	
			Agree	Disagree		
3	Gender	Male (*n =* 71)	44 (62.0)	27 (38.0)		χ^2^ = 7.41, *p =* .01
		Female (*n =* 129)	54 (41.9)	75 (58.1)		
3	Age (years)	18–24 (*n =* 26)	19 (73.1)	7 (26.9)		χ^2^ = 9.96, *p* = .04
		25–34 (*n =* 73)	30 (41.1)	43 (58.9)		
		35–44 (*n =* 49)	26 (53.1)	23 (46.9)		
		45–54 (*n =* 34)	17 (50.0)	17 (50.0)		
		55 and older (*n =* 18)	6 (33.3)	12 (66.7)		

As Table 3 details, there was evidence of a significant difference in responses between males and females with regard to the acceptability of the doctor's informing the father of paternity even if the mother does not want them to (scenario 1D—INFORM χ^2^ = 8.61, *p* = .01; scenario 2B—TELL TRUTH χ^2^ = 5.80, *p* = .05). A greater proportion of female respondents (scenario 1D—INFORM: 81.4%; scenario 2B—TELL TRUTH: 49.6%) and a lower proportion of male respondents (scenario 1D—INFORM: 64.8%; scenario 2B—TELL TRUTH: 33.8%) were of the view that this action is not ethically permissible. Additionally, in scenario 1D—INFORM, in which the father had not asked whether doctors could tell whether the boy was his son, a greater proportion of male respondents (22.5%) and a lower proportion of female respondents (8.5%) were ambivalent about the ethical permissibility of this action.

There was evidence of a difference in responses between age groups with regard to the ethical permissibility of the doctor doing what they believe to be in the best interests of the child (scenario 2F—DOCTOR DECIDES χ^2^ = 18.94, *p* = .02). A greater proportion of respondents in age groups (in years) 45–54 (64.7%) and 55 plus (61.1%) and a lower proportion of respondents in age groups 18–24 (42.3%), 25–34 (42.5%), and 35–44 (44.9%) were of the view that this action is ethically acceptable. Furthermore, a greater proportion of 18- to 24-year-olds (46.2%) were ambivalent about the ethical acceptability of this action, while ambivalence varied between 8.8% and 22.2% in respondents from all other age groups. Table 4 outlines these results.

There was evidence of a significant difference in responses between gender and between age groups with regard to agreement with routine paternity testing at birth (additional question 3—gender, χ^2^ = 7.41, *p* = .01; age χ^2^ = 9.96, *p* = .04). A greater proportion of male respondents (62.0%) and a lower proportion of female respondents (41.9%) were in agreement with this action. Furthermore, a greater proportion of 18- to 24-year-olds (73.1%) were in agreement with this action, while agreement varied between 33.3% and 53.1% in respondents from all other age groups. Table 4 outlines these results.

## Discussion

In the introduction, we noted that various contradictory guidelines have been issued regarding good practice in cases of incidental findings of misattributed paternity. Overall, our respondents deemed that the most acceptable course of action across the two settings of misattributed paternity was for the doctor to discuss the paternity results only with the mother, a course of action that was recommended in the IOM's (1994) guidelines on misattributed paternity, but that has been staunchly criticized by some bioethicists (Lucassen and Parker 2001; Lucast 2007; Ross 1996; Schroder 2009; Turney 2005; Wright et al. 2002).

That said, the responses of the participants in our study also suggest that there is a lack of consensus about the permissibility of different courses of action in cases of incidental findings of misattributed paternity. This is consistent with the disagreement among ethicists upon the moral question of whether nonmaleficence and the mother's right to confidentiality should trump the father's autonomy and interests in this context (Lucassen and Parker 2001; Lucast 2007; Ross 1996; Schroder 2009; Turney 2005; Wright et al. 2002).

How should professionals proceed in the light of such disagreement? One cannot conclude what the right course of action is from such empirical surveys of public opinion. However, since it is plausible to assume that there is scope for reasonable disagreement about which position in the academic literature best identifies the right course of action, these survey results support a pluralistic, context-specific approach. That is, several different approaches may be both ethically defensible and also defensible to members of the public who are ultimately the funders and consumers of genetic services. In such cases, professionals must make a decision when the issue of misattributed paternity arises in the context of a particular child in a particular relationship with a couple. The results of this study indicate that people do not agree about what constitutes acceptable courses of action in cases of misattributed paternity. This suggests that health professionals ought to be sensitive to people's needs, and not have a single principle-based course of action for all cases of misattributed paternity. This finding complements Wertz and Fletcher's (1991) empirical work suggesting that genetic counselors adopt a care-based rather than a principled rights-based perspective on ethical issues in the clinic. Further work would be needed to ascertain whether members of the lay public adopt a similar care-based approach, but this study offers preliminary data to motivate research in this direction.

In their analysis, Wertz and Fletcher (1991) advocate an approach in which only the mother is informed about misattributed paternity but she is urged to inform the father. However, they also suggest that when the father has expressed a desire to know, he must be told the truth. In our study, the fact that the father's inquiry as to whether the physician could ascertain the child's biological parents had a significant impact on assessments of the acceptability of disclosure suggests that lay people agree with Wertz and Fletcher (1991) that the presumed father's expression of a desire to know changes the moral landscape of a case of misattributed paternity. That said, *contra* to Wertz and Fletcher's (1991) approach, a significant finding in our study is that the majority of our respondents maintained that it should ultimately be left to the mother to decide what to do.

Wertz and Fletcher (1991) do not explain why they believe that the father's expression of a desire to know changes the moral landscape in this way, and our quantitative approach also does not reveal respondents’ grounds for this view. However, one plausible explanation for this is that the father's inquiry removes the epistemic barrier that physicians face in relation to whether or not the father himself actually wants to know this information; with this epistemic barrier in place, they may not know whether disclosure will serve his well-being or autonomy, all things considered. Alternatively, it might be claimed that if the father has expressed a desire to know, the doctor is more obviously deceiving the father if he or she does not disclose the information. However, as Ross (1996) has argued, nondisclosure in misattributed paternity amounts to deception even if the father has not expressed a desire to know. A third possibility is that a father's expressed desire to know may reflect existing doubts and suspicion on the father's part and thereby indicate that there might not be marital harmony that disclosing the truth will disrupt.

Regardless of the actual explanation underlying the data, that this factor had such a significant impact on assessments of the acceptability of disclosure suggests that our respondents did not understand the argument in favor of disclosure to derive simply from a deontological principle that the doctor–patient relationship ought to be based on trust and truth-telling (Ross 1996); if that had been the case, there would have been equal support for disclosure across the two settings. Rather, our data suggest that they understood the argument in favor of disclosure to be grounded in large part by the importance of the information to the father's autonomy.

This finding also has important implications for clinicians adopting a strategy of attempting to discuss an approach to the potential problem of an incidental finding of misattributed paternity prior to testing. Arguably, such a strategy is generally advisable when incidental findings may occur in clinical genetics (American College of Medical Genetics Board of Directors 2012). Yet in the case of nonpaternity, this may not be desirable; for instance, one argument against this general strategy in the case of nonpaternity, which Lucassen and Parker (2001) outline, is that this approach may needlessly cause distress to some couples to avoid what is only a rare occurrence. Alternatively, Lucast (2007) has argued that such a strategy fails to avoid the conflict between promoting the presumed father's autonomy and treating both partners equally when opinions diverge about which approach to incidental findings to take in the pretest consultation.

Our findings here lend some support to an approach in which the clinician is made aware of the father's desire to know information about the child's paternity (given the significant impact of this factor in our data), even if the most acceptable course of action is still ultimately to let the mother, in private consultation with the counselor, decide whether to authorize disclosure if misattributed paternity is discovered (in accordance with the majority of our respondents). Such an approach may not avoid the conflict Lucast (2007) draws attention to, but at least an awareness of the father's desire for information makes clear when such a conflict does in fact exist; if the father expresses a desire not to know this information, disclosure would plausibly not promote his autonomy, and there is thus no conflict with equality of the sort that Lucast (2007) highlights. The remaining question is whether the benefit of establishing whether the conflict exists is sufficient to outweigh the risk of distress that Lucassen and Parker (2001) discuss. Such a strategy is also congruous with our preceding observations about the lack of consensus in public opinion, and the need for a context-specific approach. However, the fact remains that the most significant contextual factor in assessments of the acceptability of disclosure to the father observed in our study was whether the father wanted to know this information; it seems that a successful context-specific approach for misattributed paternity will need a mechanism for ascertaining the father's wishes in this regard. That said, a significant remaining challenge is how this could be best assessed in practice.

A further notable finding of this study is the significant demographic differences that were not identified in Turney's (2005) study, and, conversely, our failure to replicate her finding that education levels had a significant impact on attitudes toward disclosing paternity to the father. Most strikingly in our study, men were significantly more likely to support informing the father about misattributed paternity and universal paternity testing at birth. One potential explanation for this is that there is an epistemic asymmetry between paternity and maternity; paternity is normally established on the basis of trust between the two parties of the relationship, whereas maternity is known through observing a woman carrying and giving birth to the child. As such, men, who are epistemically disadvantaged in this sense, may plausibly believe clinicians ought to remedy this asymmetry if the information is available. Interestingly, though, Lucast (2007) suggests that this same epistemic asymmetry implies that women, rather than men, are disadvantaged because they, unlike men, cannot conceal their infidelity.

In the light of Lucast's (2007) argument, we might propose two speculative hypotheses for the gender difference evident in our study. First, this might potentially reflect different views about the disadvantages that each sex faces by virtue of this epistemic asymmetry. Second, Lucast's (2007) analysis suggests an alternative but related explanation of the gender difference evident in our data. According to Lucast (2007), while we might generally agree that clinicians should give equal consideration to their clients in making decisions about disclosure in cases of misattributed paternity, whether or not this principle supports disclosure to the father depends on one's understanding of equality. On what she calls “narrow-context views,” the principle of equality requires that the mother and presumed father are treated equally as clients of the genetic counselor, but not with regard to their wider social situation. In contrast, what she calls “broad-context views” claim that equality requires taking these broader social concerns into consideration. In addition to the disadvantage related to the epistemic asymmetry discussed in the preceding, Lucast (2007) points out that the establishment of misattributed paternity may have more negative repercussions for the female than it does for males on broad-context views.

Accordingly, Lucast (2007) suggests that the narrow-context view of equality naturally aligns with the claim that information about paternity should be disclosed to the father, while the broad-context view aligns with the claim that only the woman should be consulted, due to her comparative social disadvantage. Our data might thus plausibly reflect a gender difference with regard to narrow and broad understandings of equality in genetic counseling. Regardless, the gender difference evident in our data is important, given the fact that this effect has not been observed in other lay surveys, and its potential implications for how to deal with disclosure of misattributed paternity.

This study has limitations. First, it was conducted with a relatively small sample, and should thus be viewed as providing preliminary data to direct further research. Second, due to the logistics of employing Mechanical Turk, it is possible the participant sample is not entirely representative of the general population, as access to a computer and the Internet was necessary to complete the survey. As participation in this study was voluntary, an ascertainment bias may have also occurred, in that those who participated may have had stronger views about the issues raised. As with all studies that include a questionnaire, the language used and examples given in the questionnaire may influence participants’ responses. Other courses of action could have been considered (e.g., omitting to discuss the issue of paternity), which might have received different responses.

Third, it should be acknowledged that the study was based upon the views of lay people in the United States. It is hypothesized that cultural and political contexts, along with access to affordable health care, may have significant influence upon the views regarding access to genetic and genomic information, and thus the views elicited in this study may not be representative of the views of lay people in other countries. For example, Tozzo and colleagues (2014) have illustrated that there are both differences and similarities in ethical arguments and practice in Italy compared to the United Kingdom with regard to misattributed paternity. However, in saying this, the study did raise the important point that people want health professionals to be sensitive to the needs of individuals involved, and not have a single, inflexible course of action for dealing with the incidental finding of misattributed paternity. Although we cannot say that this view would be reflected in other countries, it does present a significant viewpoint about the issue, of which clinicians and commentators in a global context should be conscious.

Fourth, we should also emphasize that participants were asked whether different possible courses of action were acceptable. Since more than one course of action can be morally acceptable in many situations, our results do not provide direct information about what lay people believe is the most morally correct answer in these situations. It cannot be ruled out, for example, that the course of action deemed acceptable by the largest number of participants was not the one that most of them thought was the morally best one. We do not, however, believe that this is a major limitation, given that for purposes of law and practice the more crucial question is whether there is general consensus about the moral permissibility of a given policy.

## Conclusion

Misattributed paternity continues to be a highly divisive issue in clinical ethics. The preliminary data we have presented in this article contribute three particularly important findings to this debate. First, in our sample, there is a lack of consensus among lay attitudes toward the moral permissibility of different courses of action that clinicians might take in cases of misattributed paternity. Although this is congruous with the lack of consensus in the clinical ethics literature, our data suggest that our respondents’ attitudes were, at least to some degree, at variance with some professional guidelines. The majority of our respondents seemed to reject the 1983 President's Commission for the Study of Ethical Problems in Biomedical and Behavioral Research position on disclosure of paternity, supporting the IOM's position that only the mother should be informed about misattributed paternity. However, the lack of consensus among our respondents and the significant influence of contextual factors suggest a case-specific approach to this issue, as Wertz and Fletcher (1991) have advocated. Such an approach would involve bringing abstract ethical principles “down to earth” (Wertz et al. 1990) by considering them in the light of concrete features of a particular case, and attempting to resolve conflicts between them through a process of wide reflective equilibrium (Davies et al. 2015). As such, while general guidelines ought to be considered, context-specific moral judgments should not be avoided.

Second, our data also suggest some of the relevant ethical considerations that should be incorporated into case-specific judgments. Our results suggest that the presumed father's desire to know information about paternity had a significant impact on lay attitudes toward acceptable practice in cases of misattributed paternity. For our respondents, this was the single most important contextual factor influencing judgments about the acceptability of different options available to doctors. This, we suggest, provides some support for pretesting consent to disclose paternity or discussion of the possibility of this incidental finding.

Finally, we also observed significant gender differences in this study. While we have provided some putative explanations for these differences, we suggest that they raise further questions that we have lacked space to address here about how such differences could be accommodated in the clinic—should we adopt broad- or narrow-context views of equality when considering epistemic asymmetries in paternity and maternity? Moreover, our data raise the question of whether the gender differences we observed among lay public attitudes might feasibly extend to genetic counselors. This would have significant ethical implications, and thus warrants further investigation.
